# Parathyroid hormone's enhancement of bones' osteogenic response to loading is affected by ageing in a dose- and time-dependent manner

**DOI:** 10.1016/j.bone.2017.02.009

**Published:** 2017-05

**Authors:** Lee B Meakin, Henry Todd, Peter J Delisser, Gabriel L Galea, Alaa Moustafa, Lance E Lanyon, Sara H Windahl, Joanna S Price

**Affiliations:** a School of Veterinary Sciences, University of Bristol, Bristol, UK; b Department of Surgery, Faculty of Veterinary Medicine, Kafr El-Sheikh University, Kafr El-Sheikh, Egypt; c Centre for Bone and Arthritis Research, Institute of Medicine, Sahlgrenska Academy at the University of Gothenburg, Gothenburg, Sweden; d Newlife Birth Defects Research Centre, UCL Great Ormond Street Institute of Child Health, UCL, London, UK

**Keywords:** Ageing, Osteoporosis, Bone, Parathyroid hormone, Mechanical loading

## Abstract

Decreased effectiveness of bones' adaptive response to mechanical loading contributes to age-related bone loss. In young mice, intermittent administration of parathyroid hormone (iPTH) at 20–80 μg/kg/day interacts synergistically with artificially applied loading to increase bone mass. Here we report investigations on the effect of different doses and duration of iPTH treatment on mice whose osteogenic response to artificial loading is impaired by age. One group of aged, 19-month-old female C57BL/6 mice was given 0, 25, 50 or 100 μg/kg/day iPTH for 4 weeks. Histological and μCT analysis of their tibiae revealed potent iPTH dose-related increases in periosteally-enclosed area, cortical area and porosity with decreased cortical thickness. There was practically no effect on trabecular bone. Another group was given a submaximal dose of 50 μg/kg/day iPTH or vehicle for 2 or 6 weeks with loading of their right tibia three times per week for the final 2 weeks. In the trabecular bone of these mice the loading-related increase in BV/TV was abrogated by iPTH primarily by reduction of the increase in trabecular number. In their cortical bone, iPTH treatment time-dependently increased cortical porosity. Loading partially reduced this effect. The osteogenic effects of iPTH and loading on periosteally-enclosed area and cortical area were additive but not synergistic. Thus in aged, unlike young mice, iPTH and loading appear to have separate effects. iPTH alone causes a marked increase in cortical porosity which loading reduces. Both iPTH and loading have positive effects on cortical periosteal bone formation but these are additive rather than synergistic.

## Highlights

• iPTH causes dramatic intra-cortical porosity in the aged mouse tibia.
• In trabecular bone, iPTH reduced some of the beneficial effects of mechanical loading.
• Mechanical loading reverses some of the deleterious effects of iPTH in cortical bone.
• The effects of iPTH and mechanical loading were additive.

## 1 Introduction

Throughout life, bones adapt their architecture to ensure that they are sufficiently robust to withstand the habitual levels of mechanical loading to which they are subjected without accumulation of excessive microdamage, or sustaining fracture. This functional adaptation, achieved by the processes of modelling and remodelling in response to the local strain environment engendered by loading, is commonly referred to as the “mechanostat” [1]. With increasing age there is failure to maintain the balance between formation and resorption with resulting progressive net bone loss [2]. Previous studies by ourselves and others have documented that bone's adaptive responsive to anabolic stimulation by mechanical loading is impaired in aged mice [3], [4], [5], [6]. It is probable that this reduced ability to respond appropriately to mechanical stimulation is a major contributor to the pathogenesis of age-related bone loss [7].

Pharmacotherapy targeted at enhancing aged bones response to mechanical loading should therefore have the potential to prevent some of the deleterious effects of ageing on bone and restore functionally-relevant structure. Intermittent administration of parathyroid hormone (iPTH) was, until recently, the only licensed anabolic treatment for osteoporosis [8].

The mechanism of action of PTH has been extensively studied in mice and other experimental models. PTH acts predominantly through its receptor, PTH1R. Previous studies have investigated both tamoxifen-inducible targeted deletion of PTH1R and constitutively active PTH receptor (caPTH1R) in osteocytes using a Dmp1-Cre. Deletion of PTH1R in osteocytes results in loss of both cortical and trabecular bone [9] although a further study documented conflicting results with a high bone mass phenotype [10]. Conversely, mice expressing caPTH1R in osteocytes had dramatically increased bone mass, but only when they also expressed the Wnt co-receptor LRP5 [11]. Furthermore, the caPTHR1 transgenic mice demonstrated greatly reduced Sost expression, the gene encoding sclerostin protein which negatively regulates Wnt signalling via LRP co-receptors. An additional study using Sost overexpressing and knockout mice indicated that reduction of sclerostin appears to contribute to some extent to the anabolic effect of PTH [12]. Sclerostin down regulation is also a necessary step in bone's anabolic response to mechanical stimulation [13] suggesting some commonality between the mechanisms of action of iPTH and mechanical stimulation.

A previous study from our laboratory demonstrated that combining iPTH and mechanical loading caused a far greater anabolic response than would have been expected from the response to either treatment alone in both cortical and trabecular bone compartments of the mouse tibia [14]. Therefore, we hypothesized that iPTH would have the potential to “sensitize” aged bone to the anabolic effects of mechanical loading and “rescue” its impaired adaptive response. However, to our knowledge, this interaction has only been studied in young female mice that have not yet displayed any age-related bone changes.

Although the effects of iPTH have been extensively studied, to our knowledge there are no studies showing the effect of different doses or duration of iPTH treatment in aged mice. Because different studies have used different doses and duration of treatment, it is difficult to compare the results presented by different groups. Thus, in this study, we aimed to determine the effect of different doses and duration of treatment with iPTH alone and the effect of iPTH on the response of aged bone to mechanical loading.

## 2 Materials and methods

### 2.1 Animals

Nineteen-month-old female C57BL/6 mice were obtained from Charles River Inc. (Margate, UK). All mice were allowed free access to water and a maintenance diet containing 0.75% calcium (EURodent Diet 22%; PMI Nutrition International, LLC, Brentwood, MO, USA) in a 12-h light/dark cycle, with room temperature at 21 ± 2 °C. All cages contained wood shavings, bedding and a cardboard tube for environmental enrichment. Female mice were housed in groups of up to five animals [15]. All procedures complied with the UK Animals (Scientific Procedures) Act 1986 and were reviewed and approved by the University of Bristol ethics committee (Bristol, UK).

### 2.2 Dose-response study

**Fig. 1:**
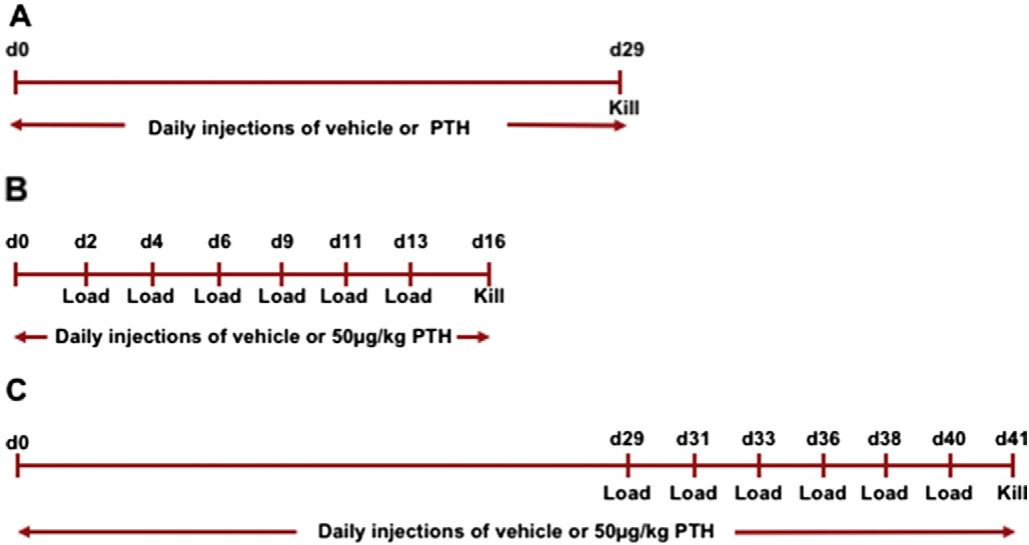
Timeline of experiments. a) In the dose response study mice were injected with 0, 25, 50 or 100 μg/kg/day by subcutaneous injection for 28 days and killed on day 29. For the loading studies, mice were injected with vehicle or 50 μg/kg/day for either b) 15 or c) 40 days with loading three times per week for the final two weeks.

Mice were divided into 4 weight matched groups (n = 8 per group) and treated with either vehicle (0.9% saline) or PTH (1–34, Cat. No H-4835, Bachem Biosciences, Switzerland) by daily subcutaneous injection. PTH was administered at 25, 50 or 100 μg/kg/day in 0.9% saline. All mice were treated for 28 days and then sacrificed (Fig. 1a). The left tibia was used for μCT scanning and the right hind limb for strain gaging (see ex vivo strain measurements).

### 2.3 Ex vivo strain measurements

To apply similar magnitudes of peak strain to all groups of mice, we first established the load:strain relationship. In each mouse, a single element strain gage (EA-06-015DJ-120, Vishay Measurement Group, NC) was bonded longitudinally to the medial aspect of the tibia at 37% of its length from the proximal end. This is the site where we have previously observed the greatest osteogenic response to axial loading [14], [16], [17], [18]. Strains were measured across a range of peak loads between 3 and 19 N, applied using the same electromagnetic loading machine used for in vivo loading (ElectroForce 3100; Bose Co., Eden Prairie, MN, USA).

### 2.4 Loading studies

Mice were treated with either vehicle or 50 μg/kg/day PTH (1–34) by daily subcutaneous injection. Mice were injected daily for 15 or 40 days for the 2-week (N = 7 and N = 6 for vehicle and iPTH treated mice respectively) and 6-week (N = 13 and N = 13 for vehicle and iPTH treated mice respectively) studies respectively. The right tibiae were subjected to external mechanical loading under isoflurane-induced anesthesia three times per week for two weeks starting from day 3 or 29 for the 2-week- and 6-week studies respectively. Left limbs were used as internal controls as previously validated (Fig. 1b-c) [16], [19]. The protocol for non-invasive loading of the mouse tibia has been reported previously [16], [20], [21]. The flexed knee and ankle joints are positioned in concave cups; the upper cup containing the knee, is attached to an actuator arm of the loading device and the lower cup to a dynamic load cell. The tibia is held in place by a 0.5 N continuous static pre-load. 40 cycles of dynamic load are superimposed with 10s rest intervals between each cycle. The protocol for one cycle consists of loading to the target strain (measured on the medial aspect of the tibia at the 37% site from the proximal end), hold for 0.05 s at the peak strain, and unloading back to the 0.5 N pre-load. From the strain gage data (see “ex vivo strain measurements”), there was no significant difference between vehicle and PTH-treated mice after 29 days of treatment in the 6-week loading study. Therefore the same dynamic load of 12.6 N and load rate of 216 Ns^− 1^ was applied to both groups of mice in the 6-week loading study.

### 2.5 High-resolution μCT analysis

Following sacrifice, lower legs were fixed in 4% PFA for 48 h at 4 °C and then stored in 70% ethanol and whole tibiae imaged using the SkyScan 1172 (Bruker, Kontich, Belgium) with a voxel size of 4.8 μm (110 μm^3^). The scanning, reconstruction and method of analysis have been previously reported [15], [22]. We evaluated the effect of iPTH on both tibiae and changes due to loading in bone volume fraction (BV/TV), trabecular thickness (Tb.Th), trabecular number (Tb.N) and trabecular separation (Tb.Sp) in the trabecular region (0.25–0.75 mm distal to the proximal physis) and cortical bone area (Ct.Ar), total cross-sectional area inside the periosteal envelope (Tt.Ar), cortical thickness (Ct.Th) and total cortical porosity (Ct.Po) at the cortical site (37% from the proximal end), according to ASBMR guidelines [23]. Previously-validated, freely available site-specificity software was used to analyze whole bones and allow comparisons of the effects of treatment across all sites in the bone as previously reported [18].

### 2.6 Fluorescent bone labelling

Fluorochrome labels were administered twice; calcein on day 1 and alizarin on day 40 (the final loading and treatment day). After μCT scanning, tibiae from the pilot study were either embedded for histology (next section) or fixed in Bürckhardt's fixative, dehydrated in increasing concentrations of EtOH, and embedded in plastic (L R White Resin; Agar Scientific, Stanstead, UK) for imaging of fluorescent bone labels. Transverse sections of 200 μm thickness sections were obtained for imaging on a confocal microscope using FITC and TRITC filters for calcein and alizarin respectively. Sectioning and imaging were performed at Pharmatest Services Ltd. (Turku, Finland).

### 2.7 Immunohistochemistry

Remaining tibiae were decalcified for 21 days in 14% EDTA with continuous agitation. The solution was changed three times per week and adequate decalcification confirmed by imaging using μCT and comparing the bone density with surrounding muscle. Bones were then processed for histology and wax embedded and sectioned transversely with 6 μm thickness. Sections corresponding to the 37% site of the tibia (measured from the proximal end and where bone formation following mechanical loading is maximal [14], [16], [17]) were stained using a standard H&E staining protocol or using the sclerostin, periostin or cathepsin K immunostaining protocol. These were as previously described [24], [25], [26]. In short, the sections were deparaffinised with xylene and rehydrated in decreasing concentrations of ethanol. Peroxidase activity was blocked with hydrogen peroxide and unspecific binding was blocked by normal rabbit or goat serum before incubation with the primary antibody; cathepsin K antibody (1 h incubation at room temperature, kindly provided as a gift from Professor Göran Andersson, Karolinska Institutet), periostin antibody (incubated over night at 4 °C, Abcam, rabbit anti-mouse/human Periostin ab14041) or sclerostin antibody (incubated over night at 4 °C, R&D systems Inc., goat anti mouse Sost AF1589). For the cathepsin K and periostin assays, a goat anti-rabbit secondary antibody (DAKO, #0432) was used; a rabbit anti-goat antibody (Dako, E0466) was used for the sclerostin assay. The signal was amplified using the vectastain Elite kit (PK 6100, Vector lab) and visualized with DAB (Vector lab, ImmPACT DAB kit SK4100). Sclerostin staining in the fibula was quantified by measuring osteocyte sclerostin stain intensity and binarizing to positive or negative staining using a grayscale cut off of 170. The fibula was analysed as this bone did not develop dramatic pores which precluded analysis of osteocyte staining in the tibia.

### 2.8 Statistical analysis

Data is presented as mean ± SEM. The effect of load on the strain measurement was assessed using linear regression. The effect loading on tibial length was assessed by paired samples *t*-test. The effect of iPTH on measures of bone mass and architecture was assessed using a one-way ANOVA with post-hoc Bonferroni correction. The effect of loading and iPTH on measures of bone mass and architecture within the two- and six-week experiment was assessed separately using two-way repeated measures ANOVAs to account for the left and right limbs being paired within each mouse. The effect of iPTH or vehicle on sclerostin expression in the fibula was assessed using an independent samples *t*-test.

Statistical comparison of Site Specificity analyses was by mixed model analysis with bone site as a fixed categorical parameter, the intervention (loading, PTH) as a fixed effect and an intervention by site interaction to account for site-specific responses. Mouse ID was included as a random effect. When the effect of the intervention was significant overall, a post-hoc Bonferroni correction was applied to identify the individual sites at which the effect was significant at p < 0.05. All statistics were performed using SPSS for Windows (version 23, IBM, Chicago).

## 3 Results

Bodyweights and tibial lengths did not change significantly in any group over the course of the study: There was an average 0.5% loss of bodyweight over 28 days in the pilot dose response study (p = 0.63), a 2.5% loss over the 17 days in the 2-week study (p = 0.31), and a 1.1% loss over 40 days in the 6-week study (p = 0.07). PTH treatment had no significant effect on tibial length in either the pilot dose response (p = 0.54), the 2-week (p = 0.22) or in the 6-week study (p = 0.54). Loading had no effect on tibial length in the 2-week (p = 0.52) or the 6-week study (p = 0.15).

### 3.1 The effect of iPTH dose on trabecular and cortical bone in the tibia

**Fig. 2:**
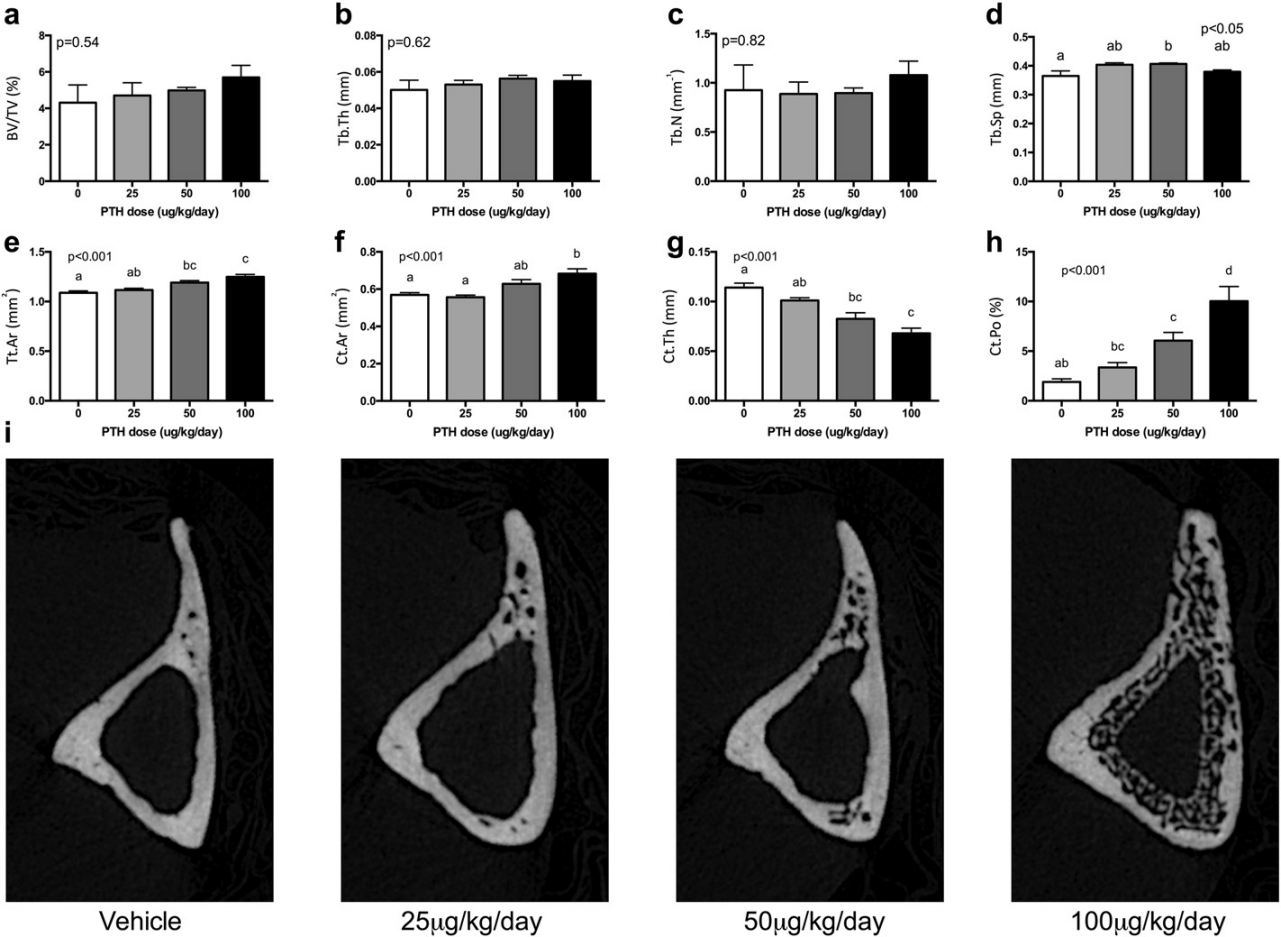
The effect of various doses of iPTH on trabecular and cortical bone mass and architecture after 28 days of treatment. Mice were administered 0, 25, 50 or 100 μg/kg/day and bone analysed by μCT. iPTH had no clear dose response effect on trabecular BV/TV (a), Tb.Th (b) or Tb.N (c) but did cause mild variation in Tb.Sp (d). Conversely there was a marked increase in Tt.Ar (e), Ct.Ar (f) and Ct.Po (h) with a corresponding decrease in Ct.Th (g) as shown in representative images (i). Analysis was by one-way ANOVA with p-values indicated on the graphs. Where significant, post-hoc Bonferroni was performed. Bars with the same letter above were not significantly different from each other.

In trabecular bone, there was no clear dose-response effect for any dose of iPTH on any of the measured parameters (see Fig. 2a–d). There was an isolated significant effect of iPTH dose on Tb.Sp due to a significant increase in Tb.Sp with 50 μg/kg/day compared to vehicle (Fig. 2d). In contrast to the small effect of iPTH dose on trabecular bone, in cortical bone the response was pronounced and followed a clear dose-response pattern (Fig. 2e–i). Tt.Ar dose-dependently increased with iPTH (p < 0.001, Fig. 2e) suggesting periosteal expansion. Ct.Ar also dose-dependently increased (p < 0.01, Fig. 2f), although only with the 100 μg/kg/day dose was a significant difference observed from vehicle in post-hoc comparisons. There was no significant effect of iPTH dose on Ma.Ar (p = 0.61). iPTH dose-dependently reduced Ct.Th (p < 0.001, Fig. 2g). This occurred due to the formation of intra-cortical pores as indicated by a dose-dependent increase in total porosity (Ct.Po) (p < 0.001, Fig. 2h).

### 3.2 iPTH induces porosity due to intracortical osteoclastic bone resorption

**Supplementary Fig. 1:**
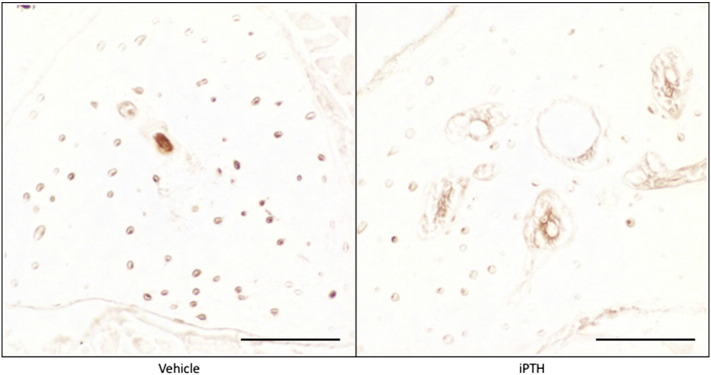
Mice were treated with vehicle or 50 μg/kg/day for 28 days. The fibula was sectioned and immunostained for sclerostin. Scale bars equals 100 μm.

**Fig. 3:**
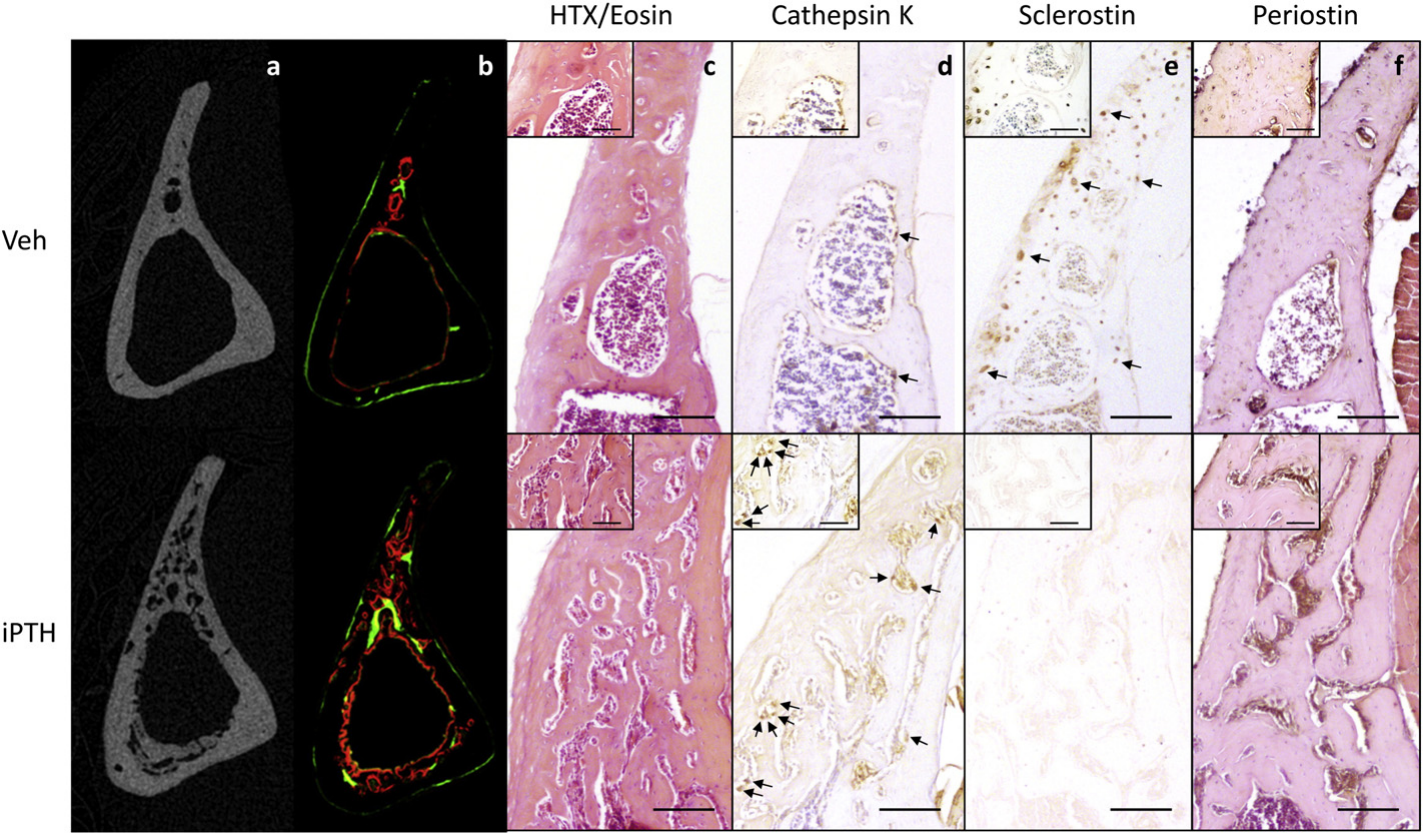
Mice were treated with vehicle or 50 μg/kg/day for 28 days and the effect on bone architecture examined using μCT (a) and fluorescent bone labels (b). Further sections of bone were stained with H&E (c) or immunostained for cathepsin K (d), sclerostin (e) or periostin (f). Large images are 10 times and insert images are 40 times magnification. Scale bars in large images equals 100 μm and in insert images 50 μm. Osteoclasts were defined as large, strongly cathepsin K stained cells on a bone surface and are indicated by arrows in figure d. Examples of sclerostin positive osteocytes are indicated by arrows in figure e.

Examination of fluorescent labels indicated the intra-cortical pore formation identified on μCT (Fig. 3a) was a dynamic process that occurred within the study period during which iPTH was administered (Fig. 3b). Sections of tibial cortex from the 37% site were also stained using H&E in addition to immunohistochemical stains for cathepsin K, sclerostin and periostin. The H&E stain revealed the intracortical pores to contain large multinucleated cells with the features of osteoclasts (Fig. 3c) that stained positive for cathepsin K (Fig. 3d). This process was visible, although not so dramatic, after two weeks of iPTH treatment. Two and six weeks of iPTH treatment resulted in a decrease in the number of osteocytes staining positive for sclerostin (Fig. 3e). Quantification of osteocytic sclerostin staining in the fibula demonstrated that this decrease was statistically significant (vehicle 49 ± 7%, iPTH 29 ± 4%, p = 0.03, Supplementary Fig. 1). Periostin immunohistochemical staining documented strongly positive cells within the intracortical pores (Fig. 3f) which, combined with the fluorescent images, is highly suggestive of a dynamic remodelling process in response to iPTH involving osteoclastic bone resorption and osteoblastic bone formation.

### 3.3 Ex vivo strain gaging

**Fig. 4:**
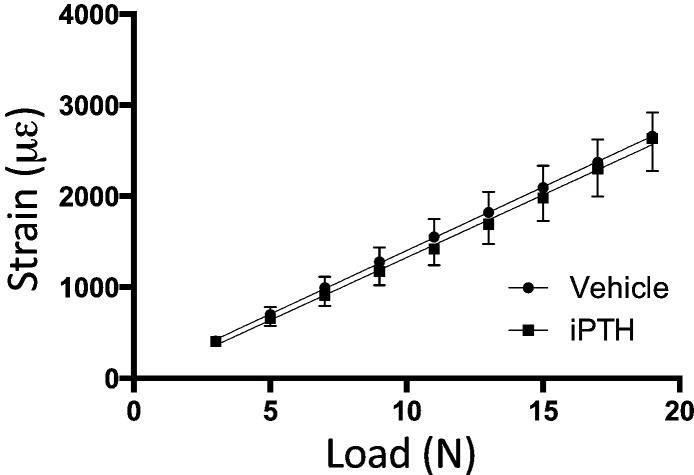
The effect of iPTH on the load strain relationship. Tibial stiffness was unaffected by treatment with iPTH.

To determine whether treatment with 50 μg/kg/day iPTH for 4 weeks altered the load:strain relationship, strain gages were attached to the medial surface of the right tibia at the 37% site. Applying various loads to the tibiae and measuring strain revealed a clear linear relationship. The gradient of both regression lines was significantly different from zero (p < 0.001). There was no significant difference between the strains engendered by loading when the vehicle and iPTH-treated bones were compared (p = 0.92, Fig. 4). Linear regression analysis allowed calculation of the loads required to apply 1800 με (11.8 N) and 1750 με (12.6 N) at the start of the 2-week- and 6-week studies respectively.

### 3.4 The effect of duration of iPTH treatment on trabecular bone; reversal of the effect of mechanical loading on trabecular bone in the proximal tibia

**Fig. 5:**
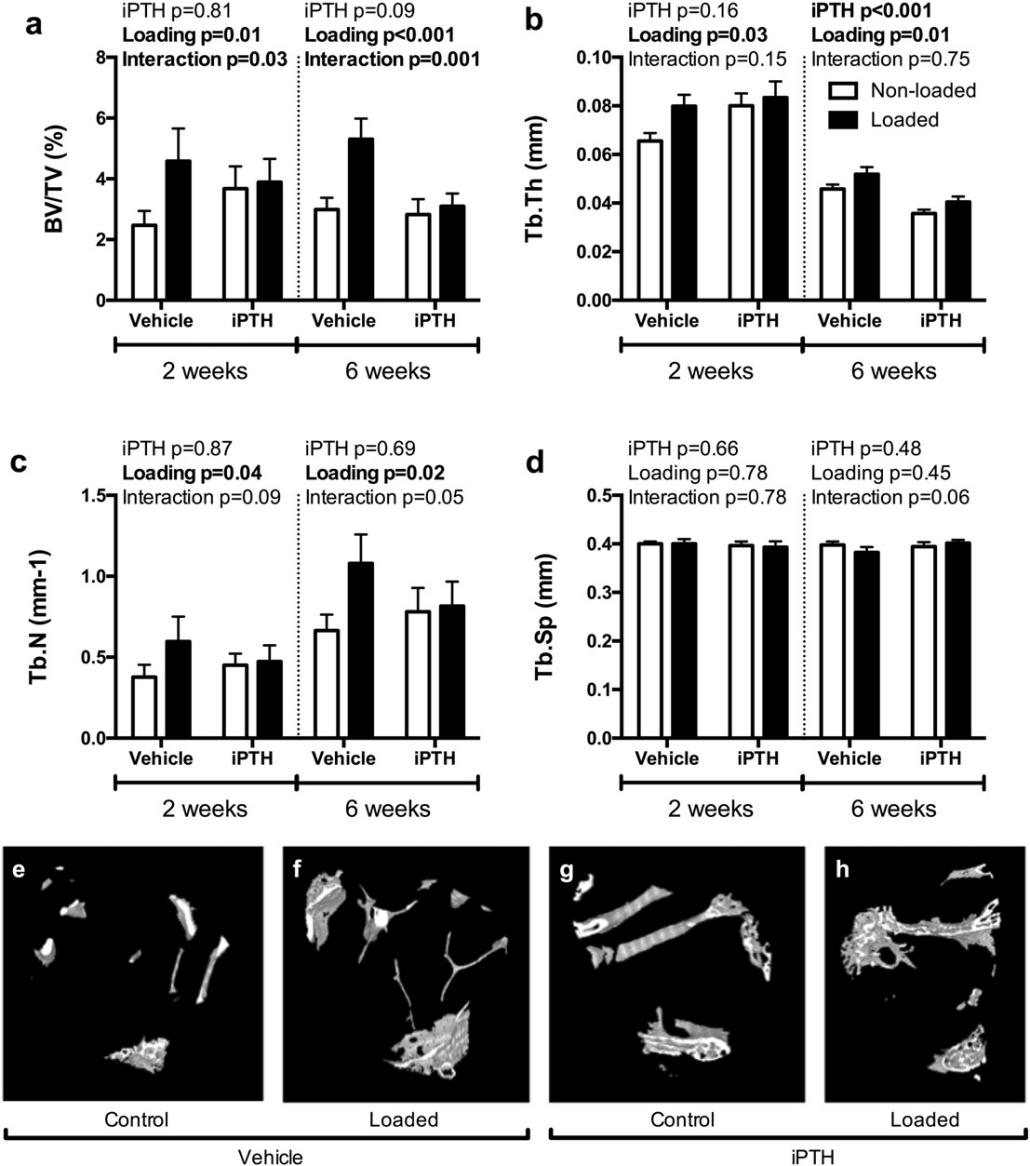
The effect of 2- and 6-weeks of treatment with vehicle or 50 μg/kg/day iPTH on the response to loading for trabecular BV/TV (a), Tb.Th (b), Tb.N (c) and Tb.Sp (d). Representative 3D reconstructions showing the effect of 6-weeks iPTH on the response to loading in the region of trabecular bone analysed by μCT (e-h). P-values represent results of the two-way repeated measures ANOVA for the 2- and 6-week loading experiments for the effect of PTH, loading and their interaction. Significant results are indicated in bold.

In trabecular bone, there was a significant interaction between the effects of iPTH and mechanical loading on BV/TV, both in the 2-week and 6-week studies (Fig. 5a). In both experiments, BV/TV was significantly increased by 2 weeks of mechanical loading in vehicle-treated mice (+ 84% and + 77% respectively), but this effect was abrogated by treatment with iPTH (Fig. 5a). Loading increased Tb.Th in the two experiments (+ 22% and + 15% respectively). While 2-week iPTH treatment had no significant effect on Tb.Th, 6-week treatment with iPTH significantly decreased Tb.Th (− 22%, p < 0.001), with no significant interaction in either study (Fig. 5b). The loading induced increase in Tb.N (+ 52% and + 62% for the two experiments respectively, Fig. 5c) was depleted with concurrent iPTH treatment although there was no significant effect of iPTH or interaction. A significant interaction was also detected between loading and iPTH on Tb.N when the mice were pre-treated with iPTH for four weeks prior loading. There was no effect of loading or iPTH on Tb.Sp (Fig. 5d).

### 3.5 The effect of duration of iPTH treatment and loading on cortical bone were additive

**Fig. 6:**
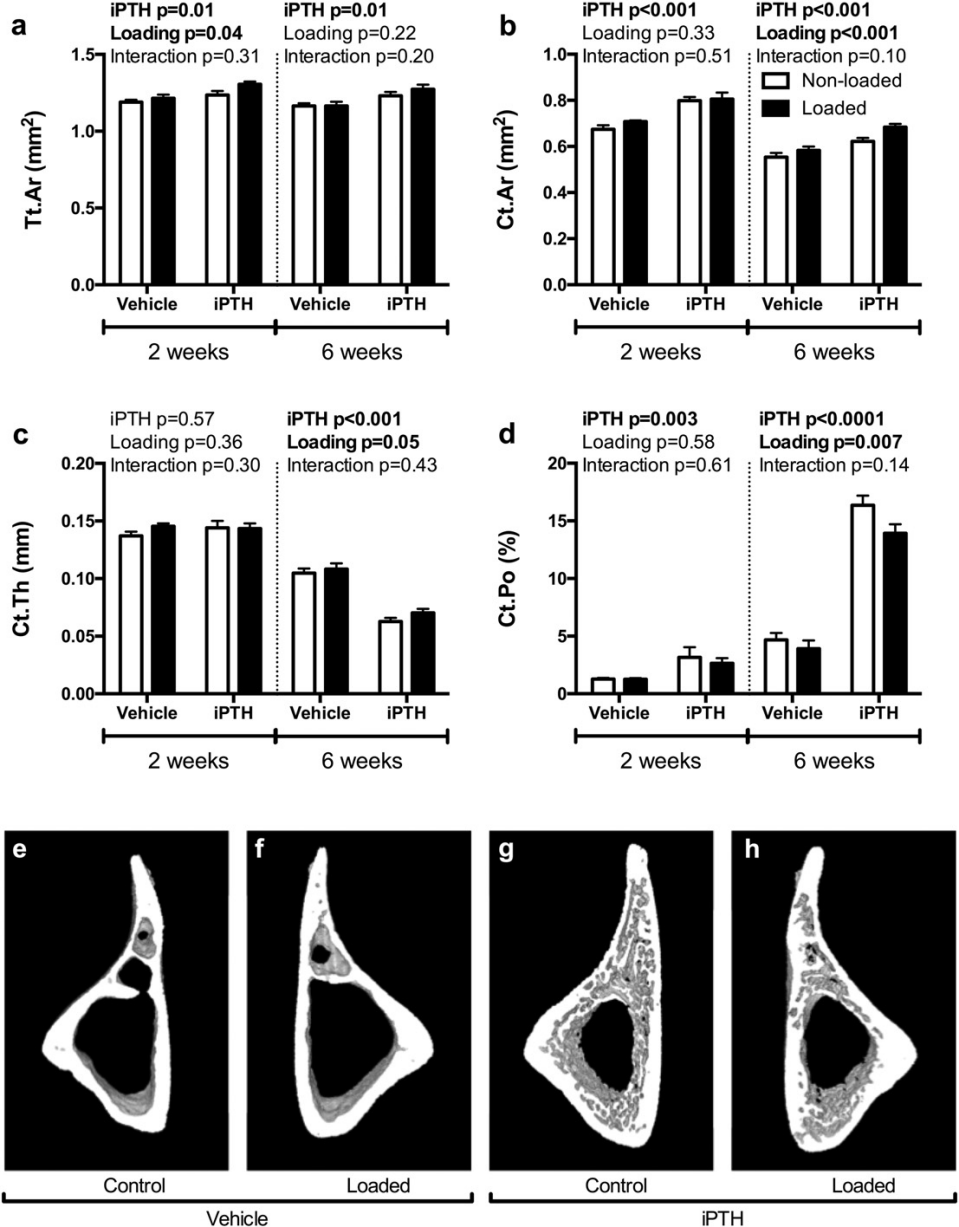
The effect of 2- and 6-weeks of treatment with vehicle or 50 μg/kg/day iPTH on the response to loading for Tt.Ar (a), Ct.Ar (b), Ct.Th (c) and Ct.Po (d). Representative 3D reconstructions showing the effect of 6-weeks iPTH on the response to loading in the region of cortical bone analysed by μCT (e-h). P-values represent results of the two-way repeated measures ANOVA for the 2- and 6-week loading experiments for the effect of PTH, loading and their interaction. Significant results are indicated in bold.

In cortical bone, loading alone increased Tt.Ar in the two- but not six-week experiment (Fig. 6a). As expected from the pilot dose-response study, iPTH increased Tt.Ar in the two- and six-week study (p = 0.01, Fig. 6a). Loading alone increased Ct.Ar (5% and 6% respectively, Fig. 6b), although this was only significant in the six-week experiment (P < 0.001). Ct.Ar was significantly increased by iPTH treatment in both experiments (+ 18% and + 13% for the two- and six-week studies respectively), although there was no interaction between loading and iPTH treatment in either study. Two weeks of concurrent iPTH treatment did not affect Ct.Th (Fig. 6c), but six weeks iPTH treatment lead to a large decrease in Ct.Th (p < 0.001) while loading caused a mild, but significant, increase (Fig. 6c). Two weeks of iPTH treatment increased Ct.Po by 1.5-fold, while a further four weeks of iPTH treatment with iPTH caused a dramatic increase in Ct.Po (2.5-fold), which was partially decreased by mechanical loading (Fig. 6d). There was no significant interaction detected between iPTH and mechanical loading for any of the measured cortical parameters suggesting the combined effects were additive or independent rather than synergistic.

### 3.6 The additive effects of iPTH and loading were observed across the length of the tibia

**Fig. 7:**
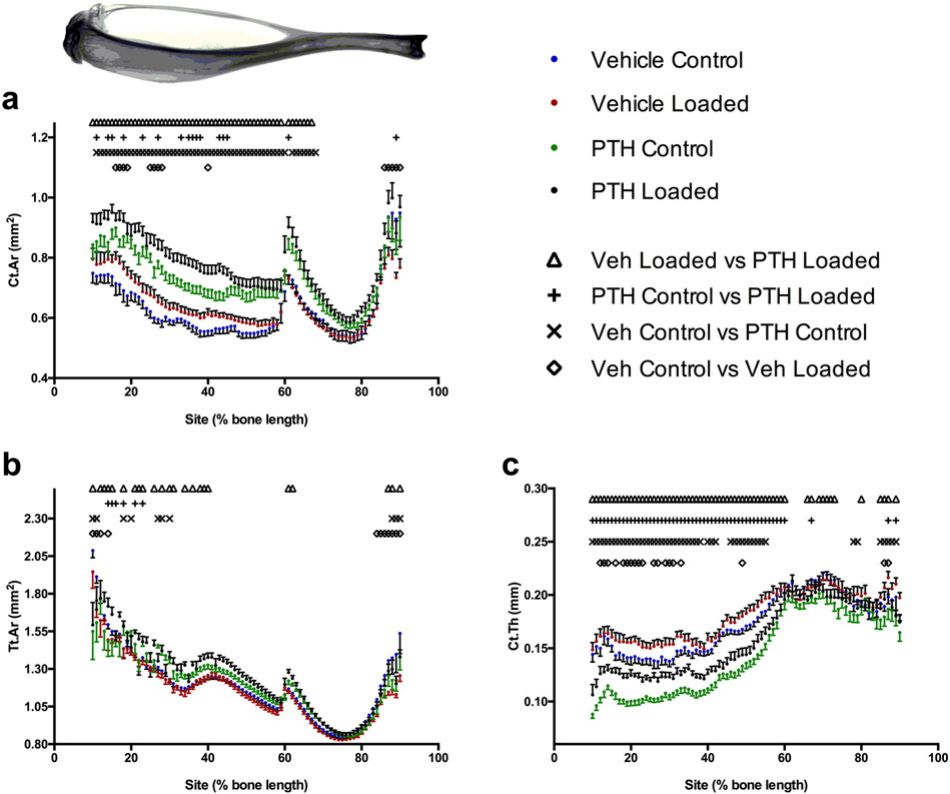
The combination of iPTH and loading additively increased Ct.Ar (a) and Tt.Ar (b) predominantly over the proximal half of the tibia. iPTH, but not loading, increased Ct.Ar (a) distal to the tibia/fibula junction (approximately 60% of the tibial length). iPTH decreased, whereas loading increased Ct.Th (c) in the proximal tibia.

A unique feature of mechanical loading as an osteoanabolic stimulus is its ability to target bone formation to functionally relevant sites. Systemic treatments such as PTH which interact with the mechanostat also have the potential to exert site-specific effects. Therefore, we used the previously-described site-specificity analysis software, [18] to map the effects of iPTH and loading on Ct.Ar, Tt.Ar and Ct.Th across the entire length of bones from the six-week iPTH study (Fig. 7). Loading predominantly increased Ct.Ar and Ct.Th site-specifically in the proximal 50% of the tibia, with minimal effects on Tt.Ar in vehicle-treated mice. iPTH increased Ct.Ar over the proximal 70% of the tibia while reducing Ct.Th in the proximal 50%. Loading of iPTH-treated mice increased Tt.Ar and Ct.Ar in sites within the proximal 30–50% of the tibia and also increasing Ct.Th over the proximal 50% of the tibia. This implies that the effects of combining iPTH and loading are additive across the proximal half of the tibia in agreement with the finding at the 37% cortical site (Fig. 7).

## 4 Discussion

Although the mechanisms of action of PTH have been extensively studied, the optimal experimental conditions to achieve maximum effects in mouse bones have not been reported. Thus in different studies, different doses and durations of treatment are used and their effects at different skeletal sites of interest analysed, making comparisons between studies difficult. Here we report the effect of four doses and two durations of treatment with iPTH alone and the effect of iPTH on the response of aged rather than young bone to artificial loading. Our data show that in old mice responses to iPTH and loading differ in both their individual and combined effects to those we previously reported in young mice [14].

Analysis of the effects of different doses of iPTH on cortical and trabecular bone in the tibia of aged mice demonstrated a dose-dependent effect of iPTH on Tt.Ar and Ct.Ar. This was expected as iPTH has previously been reported to be anabolic on both periosteal and endosteal surfaces of cortical bone in the distal femur [27]. In contrast, we found no effect of iPTH alone on trabecular bone in the proximal tibia despite being previously reported to be anabolic in the trabecular bone of the L5 vertebra [27]. This may reflect the lower trabecular content of the proximal tibia in aged mice compared with the vertebrae. An unexpected finding in the present study was the dramatic intra-cortical remodelling observed in the tibial cortex, particularly with the higher doses of iPTH in aged mice. To our knowledge, this finding has not been reported with intermittent administration of PTH in mice despite other studies using similar doses [14], [27]. This difference could reflect the different ages of mice and sites of bone analysed. The result is similar to the phenotype previously reported in the caPTH1R-Dmp1 mice which was shown to exhibit periosteal and endosteal bone formation with marked increases in intracortical remodelling and cortical porosity [28]. Although the PTH in our study was administered by daily subcutaneous injection, it is possible that the effects of PTH in aged mice are prolonged leading to a phenotype more similar to that observed with continuous activation of PTHR1.

The intracortical pore formation in the aged mice in our current study appears to be a dynamic process, cathepsin K staining demonstrating an influx of osteoclasts in response to iPTH. This increased number of osteoclasts indicates that the resorption is by osteoclasts rather than any other process such as osteocytic osteolysis [29] and is consistent with PTH's known ability to stimulate bone resorption by enhancing osteoclastogenesis [30]. The presence of periostin-positive cells within the pores and evidence of bone mineral deposition (from fluorescent labelling) indicates concomitant osteoblastic bone formation. This suggests that iPTH may be stimulating simultaneous bone resorption and formation.

We had expected from our previous study in young mice [14] that iPTH would interact with mechanical loading resulting in proportionally larger increases in bone mass and architecture than those previously observed in aged mice [3]. Contrary to our expectation, in trabecular bone iPTH abrogated some of the beneficial effects of mechanical loading; for example on BV/TV and trabecular number. The proximal tibia of aged mice contains very few trabeculae and proportionally fewer than bones such as the vertebra. Nevertheless, the response in aged mice is different from that observed in young mice using the same loading model. This emphasises the importance of being careful when extrapolating responses in young animals to those in old ones. This may be particularly true in the case of PTH as we have recently reported basal and loading-responsive transcriptomic differences between tibiae of young and aged mice [31]. These include Wnt signalling and bioenergetics processes, both of which are involved in the anabolic effects of PTH [32], [33].

In cortical bone, mechanical loading had the same beneficial effects as those previously reported such as a significant increase in cortical area and thickness [3], [6]. Some of the potentially deleterious effects of iPTH in cortical bone, such as the increase in intracortical porosity and decrease in cortical thickness, were partially rescued by mechanical loading. This is consistent with previously published data. For example, genetic knock down of the BMP1Ra increases cortical porosity which is partially rescued by increased exercise [34].

Further elucidation of the different mechanisms of action of iPTH and mechanical loading is likely to be informative. In young mice, when iPTH and loading were combined there was a potentially synergistic response suggesting some commonality, or at least complementarity, between the cellular mechanisms underlying the responses [14]. This we hypothesized to be mediated by sclerostin since both loading and iPTH are known to result in its downregulation and therefore increased Wnt signalling [11], [24]. However, in aged mice although both loading [3] and PTH (this study) cause sclerostin down-regulation there does not appear to be any evidence of interaction between the two responses in terms at least of (re)modelling. This is consistent with a recent study showing that downregulation of Sost mRNA is required for the anabolic effects of loading, but not iPTH in young adult mice [35]. Thus it may be that deficiencies in the responses of aged mice to iPTH are either independent of sclerostin or occur down-stream of its down-regulation, such as in the recruitment or proliferation of osteoblast cells [36]. The protein-level down-regulation of sclerostin with iPTH was sustained up to at least six weeks of treatment in the present study, but this did not ‘rejuvenate’ the magnitude of bone's adaptation to mechanical loading to levels expected in young mice. This suggests that the age-related impairment of bone's adaptation to loading is not entirely explained by more transient down-regulation of sclerostin [37] impairing sustained Wnt signalling activation.

Osteoblast recruitment and sclerostin regulation are site-specifically influenced by loading [3], [24], potentially-explaining the apparent restriction of osteogenic responses to axial loading to the proximal half of the mouse tibia. The ability of the distal tibia to increase in mass is demonstrated by the significant increase in Ct.Ar below the tibia/fibula junction following iPTH treatment independently of loading, as identified by site-specificity analysis. This analysis provides meaningful additional information and precludes overly-simplistic conclusions drawn from analyses of individual sites. For example, if the main analyses of this study had focused on the tibial mid-shaft (50% site), no response to loading would have been observed and the response to PTH would not have appeared meaningfully increased by loading. Site-specificity analysis therefore represents an important experimental refinement that should prevent erroneous interpretations of experiments based on results from single sites.

In summary, in aged mice there was only a small effect of iPTH alone on trabecular bone, which was independent of dose although treatment (50 μg/kg/day) for six weeks had a stronger effect than two weeks. In trabecular bone, iPTH and loading have no positive interaction, indeed iPTH appears to reduce the positive effects of loading on trabecular number. In contrast, in cortical bone iPTH had a positive osteogenic dose and time-dependent effect. However, in aged mice, unlike in young ones, this effect appears to act independently from the osteogenic effect of loading there being no interaction between the two. Thus the positive interactions between the osteogenic effects of loading and iPTH seen in young animals are not reproduced in old ones where they could therapeutically be most useful. Indeed in trabecular bone the positive interactions seen in young animals were replaced with negative ones in old ones, iPTH inhibiting loading-induced increases in BV/TV. These data reinforce the caution that must be exercised when extrapolating from young populations to old ones.

The following are the supplementary data related to this article.

**Supplementary Fig. 2:**
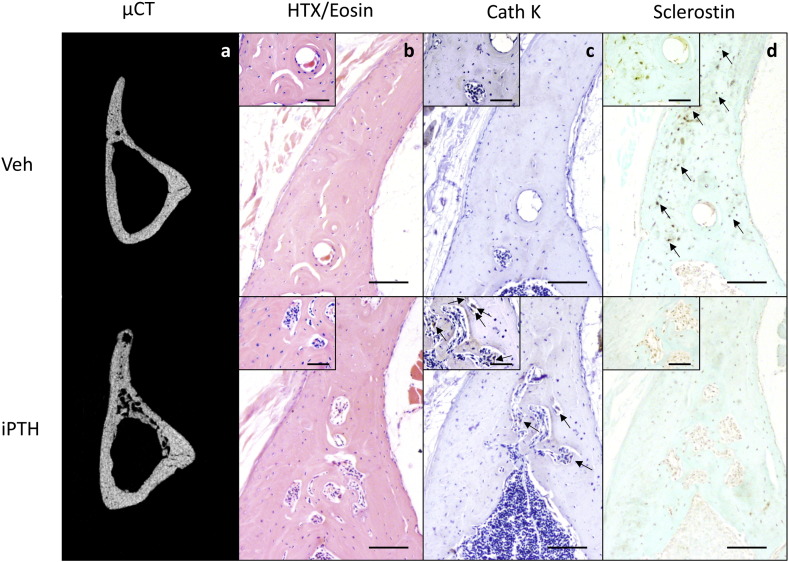
Mice were treated with vehicle or 50 μg/kg/day for 14 days and the effect on bone architecture examined using μCT (a). Further sections of bone were stained with H&E (b) or immunostained for cathepsin K (c) or sclerostin (d). Large images are 10 times and insert images are 40 times magnification. Scale bars in large images equals 100 μm and in insert images 50 μm. Osteoclasts were defined as large, strongly cathepsin K stained cells on a bone surface and are indicated by arrows in figure c. Examples of sclerostin positive osteocytes are indicated by arrows in figure d.

## Funding

GLG and LBM were supported by Wellcome Veterinary Intercalated Training Fellowships (088560/Z/09/Z and 092045/Z/10/Z respectively) and GLG is currently supported by a Wellcome Postdoctoral Clinical Training Fellowship (107474/Z/15/Z). AM was supported by the Egyptian Ministry of Higher Education. SHW was funded by a European Union's Horizon 2020 research and innovation programme under the Marie Skłodowska-Curie grant agreement No. 657178, the Swedish Research Council (2013-8252) and the ALF/LUA research grant in Gothenburg (161891).

## Disclosures

All authors state they have no conflict of interest.
